# Sex differences in immunotherapy outcomes and tumor-infiltrating immune cell profiles in patients with advanced renal cell carcinoma

**DOI:** 10.1007/s00262-024-03876-2

**Published:** 2025-01-03

**Authors:** Hiroki Ishihara, Hironori Fukuda, Yukihiro Mizoguchi, Makiko Yamashita, Kazunori Aoki, Ryo Ishiyama, Takashi Ikeda, Yuki Nemoto, Hiroaki Shimmura, Yasunobu Hashimoto, Kazuhiko Yoshida, Toshihito Hirai, Junpei Iizuka, Daisuke Tokita, Tsunenori Kondo, Yoji Nagashima, Toshio Takagi

**Affiliations:** 1 Department of Urology, Tokyo Women’s Medical University, 8-1 Kawada-Cho, Shinjuku-Ku, Tokyo, Japan; 2 Department of Immune Medicine, National Cancer Center Research Institute, 5-1-1, Tsukiji, Chuo-Ku, Tokyo, Japan; 3 Division of Cancer Immunotherapy Development, Center for Advanced Medical Development, The Cancer Institute Hospital of Japanese Foundation for Cancer Research, 3-8-31, Ariake, Koto-Ku, Tokyo, Japan; 4 Department of Urology, Saiseikai Kazo Hospital, Kamitakayanagi, Kazo, Saitama 1680 Japan; 5 Department of Urology, Tokyo Women’s Medical University Adachi Medical Center, 4-33-1 Kouhoku, Adachi-Ku, Tokyo, Japan; 6 Department of Urology, Jyoban Hospital, Uenodai 57, Joban Kamiyunagayamachi, Iwaki, Fukushima Japan; 7 Department of Urology, Saiseikai Kawaguchi General Hospital, 5-11-5 Nishikawaguchi, Kawaguchi, Saitama Japan; 8 Department of Surgical Pathology, Tokyo Women’s Medical University, 8-1 Kawada-Cho, Shinjuku-Ku, Tokyo, Japan

**Keywords:** Kidney cancer, metastatic renal cell carcinoma, tumor microenvironment, tumor infiltrating immune cell

## Abstract

Sex differences in the outcomes of advanced renal cell carcinoma (RCC) treated with immune checkpoint inhibitors (ICIs) and the profiles of tumor-infiltrating immune cells (TIICs) remain unclear. We retrospectively evaluated data from 563 patients with RCC receiving systemic therapy, including first-line dual ICI combinations (i.e., immunotherapy [IO]-IO), combinations of ICIs with tyrosine kinase inhibitors (TKIs) (i.e., IO-TKI), TKI monotherapy, and subsequent nivolumab monotherapy. Survival and tumor response were compared between the sexes in each treatment group, and TIIC profiles were compared using 116 RCC tumor samples analyzed by flow cytometry. Progression-free survival (PFS) was shorter in female than in male patients in the IO-IO (*p* = 0.0227) and nivolumab monotherapy (*p* = 0.0478) groups. Furthermore, sex remained an independent factor for shorter PFS after adjusting for covariates in the IO-IO (*p* = 0.0340) and nivolumab monotherapy (*p* = 0.0322) groups. In contrast, PFS was not significantly different between sexes in the IO-TKI or TKI monotherapy groups (*p* > 0.05). Overall survival and objective response rates were not significantly different between the sexes in any of the treatment groups (*p* > 0.05). Some TIIC populations, including that of CD8 + T cells (*p* = 0.0096), decreased to a greater extent in female than in male patients in the advanced-stage population. In conclusion, the effectiveness of ICIs on PFS was lower in female patients than in male patients, potentially because of the different profiles of the immune microenvironment, particularly the decreased number of CD8 + T cells in females.

**Supplementary Information:**

The online version contains supplementary material available at 10.1007/s00262-024-03876-2.

## Introduction

Immunotherapy (IO) has emerged as a pivotal player in the systemic treatment of advanced cancers, including renal cell carcinoma (RCC). Nivolumab, an antibody that inhibits the programmed cell death protein 1 (PD-1), is an immune checkpoint inhibitor (ICI) that was initially approved for systemic therapy following the failure of prior tyrosine kinase inhibitors (TKIs) [1]. Thereafter, several therapeutic regimens comprising ICIs targeting immune checkpoints such as PD-L1 or CTLA-4 have been implemented for RCC treatment. Currently, dual ICI combinations (i.e., IO-IO) and combinations of ICIs with TKIs (i.e., IO-TKI) are endorsed as the standard of care and first-line therapy, a recommendation based on evidence from pivotal, randomized, phase III clinical trials [2–6].

The implementation of ICIs has significantly improved the outcomes of advanced RCC [7–9], although most patients eventually experience disease progression when they do not achieve durable responses. Therefore, to provide more effective treatment, biomarkers for patient selection or prognostic prediction are urgently required. However, we have not identified biomarkers that can be utilized in routine clinical practice, and this remains an unmet need [10].

The effects of sex on ICI treatment outcomes have been extensively discussed. A systematic review and meta-analysis using clinical trial data from patients with multiple cancer types and ICI classes showed that both male and female patients showed higher efficacy of ICIs than control treatments, although the magnitude of the benefits was lower in female patients than in male [11]. A systematic review and meta-analysis, including clinical trials with a large number of immunotherapy agents and an updated search, showed no difference in immunotherapy efficacy between sexes [12]. In contrast, in studies using exclusive cohorts of patients with non-small cell lung cancer or melanoma, female patients derived fewer benefits from ICIs than male patients [13, 14]. Thus, the effects of sex on ICI outcomes remain controversial. Most importantly, in the aforementioned two meta-analyses, the population of RCC was quite limited in the entire cohort (only two trials, CheckMate 025 and CheckMate 214, were included), which made it difficult to interpret the impact of sex on the outcomes of ICIs in patients with RCC [11, 12].

Using real-world data, we previously reported that female patients had shorter progression-free survival (PFS) than male patients when treated with nivolumab plus ipilimumab combination therapy (i.e., IO-IO) or nivolumab monotherapy [15]. However, it remains unclear whether the association of sex with ICI treatment outcomes is specific to ICIs, and additional data regarding the prognostic impact of sex in TKI monotherapy or IO-TKI combination therapy are still needed. In addition, to gain a deeper understanding of the mechanism underlying sex differences in the effectiveness of ICIs, it is necessary to examine the profiles of the tumor immune microenvironment, including tumor-infiltrating immune cells (TIICs) according to sex; however, such investigations are still limited.

In this context, we retrospectively analyzed the association between sex and the effectiveness of systemic therapy for RCC across various treatment groups: IO-IO and IO-TKI combination therapies, nivolumab monotherapy, and TKI monotherapy. In addition, we investigated the differences in the profiles of TIICs in RCC tumor samples between sexes.

## Patients and methods

### Patient and RCC tumor samples

All clinicopathological and laboratory data were obtained from electronic databases and patient medical records. A total of 633 patients with advanced RCC—who received at least one administration of systemic therapy including the IO-IO, IO-TKI, and TKI monotherapy as first-line therapy, and nivolumab monotherapy as subsequent therapy between April 2008 and August 2023—were identified from across the following five institutions: Tokyo Women’s Medical University, Tokyo Women’s Medical University Adachi Medical Center, Saiseikai Kawaguchi General Hospital, Saiseikai Kazo Hospital, and Jyouban Hospital. Of these, 70 patients were excluded for the following reasons: those who received adjuvant therapy (n = 10), those whose International Metastatic RCC Database (IMDC) or baseline clinicopathological data were lacking (n = 33), and those that had inadequate follow-up periods (< 2 months) (n = 27). The remaining 563 patients were included in the study.

In addition, we collected 116 RCC tumor samples obtained via radical or partial nephrectomy from two institutions—Tokyo Women’s Medical University and Tokyo Women’s Medical University Adachi Medical Center. Histopathological diagnoses were made by a certified and experienced pathologist (Y. N.) based on the 2016 WHO classification [16].

The study protocol was approved by the Institutional Ethics Review Board of Tokyo Women’s Medical University (ID: 2020-0009 and 382), and tumor samples were obtained with written informed consent. This study was conducted in accordance with the guidelines of the 1964 Declaration of Helsinki and its later amendments.

### Clinical outcomes

PFS and overall survival (OS) after the initiation of each treatment were assessed. In addition, the objective response rate (ORR) was assessed during treatment. The best overall response and ORR in measurable targeted lesions were determined using the RECIST criteria (version 1.1) [17].

To assess tumor responses to treatment, post-treatment follow-up computed tomography (CT) scans of the chest, abdomen, and pelvis were conducted at regular intervals of 4 to 12 weeks, depending on the patient’s condition. Magnetic resonance imaging (MRI), positron emission tomography (PET)/CT, and brain scans were performed as necessary. Treatments were continued until radiographic or clinical disease progression was observed, or intolerable adverse events occurred.

### Tumor infiltrating immune cells analyzed by flow cytometry

RCC tumor samples were received within 1 h of nephrectomy and immediately dissociated into single cells by mincing in Roswell Park Memorial Institute (RPMI) medium or RPMI 1640. The single-cell suspension was then washed, passed through 40 μm cell strainers, suspended in CELL BANKER 1 (Nippon Zenyaku Kogyo, Co., Ltd., Fukushima, Japan), and stored at − 80 °C until flow cytometry. Cryopreserved cells were thawed and incubated with Fixable Viability Dye eFluor 506 (Thermo Fisher Scientific, Waltham, Massachusetts, USA) for staining of dead cells for 30 min at 4 °C, followed by staining with mAbs (Supplementary Table 1) for 30 min at 4 °C. After washing, data were acquired on a LSRFortessa™ (BD Biosciences, Franklin Lakes, NJ, USA) and analyzed using FlowJo software (BD Biosciences).

### Statistical analysis

Continuous variables were analyzed using the Mann–Whitney U test, and categorical variables were analyzed using Fisher’s exact test. PFS was calculated from “treatment initiation until disease progression or death, whichever occurred first.” The OS was calculated from “treatment initiation to death due to any cause.” Patients lost to follow-up were censored at the time of last contact. Survival data were obtained until the end of November, 2023. Survival was calculated using the Kaplan–Meier method and compared using the log-rank test. Univariate and multivariate analyses using Cox proportional hazard regression models were used to identify the factors associated with survival. Risks are expressed as hazard ratios (HRs) with 95% confidence intervals (CIs). All analyses were performed using JMP software (version 17; SAS Institute Inc., Cary, NC, USA), and statistical significance was set at *p* < 0.05.

## Results

### Patient characteristics

Of a total of 563 patients, 99 (18%), 71 (13%), 117 (21%), 276 (49%) were treated with the IO-IO combination therapy, IO-TKI combination therapy, nivolumab monotherapy, and TKI monotherapy, respectively. The patient characteristics based on sex are summarized in Table 1. There were no significant differences in age; histopathology; IMDC risk; treatment line; and lung, bone, liver, and lymph node metastasis status between the sexes in the IO-IO combination, IO-TKI combination, and TKI monotherapy groups, respectively (all *p* > 0.05). In the nivolumab monotherapy group, female patients were frequently older (≥ 65 years-old) than male patients (*p* = 0.0392), while other factors were not significantly different between the sexes (*p* > 0.05).

**Table 1:** Patient characteristics of each treatment group categorized by sex

	IO-IO (n = 99)			IO-TKI (n = 71)			Nivolumab (n = 117)			TKI (n = 276)		
	Male (n = 71)	Female (n = 28)	*p*	Male (n = 52)	Female (n = 19)	*p*	Male (n = 83)	Female (n = 34)	*p*	Male (n = 199)	Female (n = 77)	*p*
Age, in years ≥ 65 (ref. < 65)	34 (48%)	16 (57%)	0.505	33 (63%)	12 (63%)	1.000	43 (52%)	25 (74%)	0.0392	96 (48%)	42 (55%)	0.421
Histopathology Clear-cell RCC (ref. non-clear-cell RCC/ unknown)	49 (69%)	21 (75%)	0.630	35 (67%)	13 (68%)	1.000	69 (83%)	28 (82%)	1.000	153 (77%)	57 (74%)	0.639
IMDC risk Favorable Intermediate Poor	0 49 (69%) 22 (31%)	0 16 (57%) 12 (43%)	0.348	11 (21%) 29 (56%) 12 (23%)	2 (11%) 14 (74%) 3 (16%)	0.378	6 (7%) 55 (66%) 22 (27%)	5 (15%) 17 (50%) 12 (35%)	0.211	24 (12%) 131 (66%) 44 (22%)	15 (19%) 47 (61%) 15 (19%)	0.281
Treatment line Second (ref. third or more later)	N.A	N.A		N.A	N.A	N.A	51 (61%)	20 (59%)	0.837	N.A	N.A	N.A
Lung metastasis Presence (ref. absence)	44 (62%)	19 (68%)	0.648	35 (67%)	10 (53%)	0.278	55 (66%)	18 (53%)	0.210	134 (67%)	46 (60%)	0.261
Bone metastasis Presence (ref. absence)	16 (23%)	7 (25%)	0.796	11 (21%)	4 (21%)	1.000	21 (25%)	11 (32%)	0.496	46 (23%)	18 (23%)	1.000
Liver metastasis Presence (ref. absence)	8 (11%)	6 (21%)	0.210	9 (17%)	3 (16%)	1.000	14 (17%)	5 (15%)	1.000	28 (14%)	10 (13%)	1.000
Lymph node metastasis Presence (ref. absence)	23 (32%)	11 (39%)	0.639	13 (25%)	4 (21%)	1.000	28 (34%)	11 (32%)	1.000	59 (30%)	21 (27%)	0.768

*IO* immunotherapy, *TKI* tyrosine kinase inhibitor, *RCC* renal cell carcinoma, *IMDC* international metastatic renal cell carcinoma database consortium, *N.A.* not applicable, *ref* reference

### Efficacy profile based on the sex

**Fig. 1:**
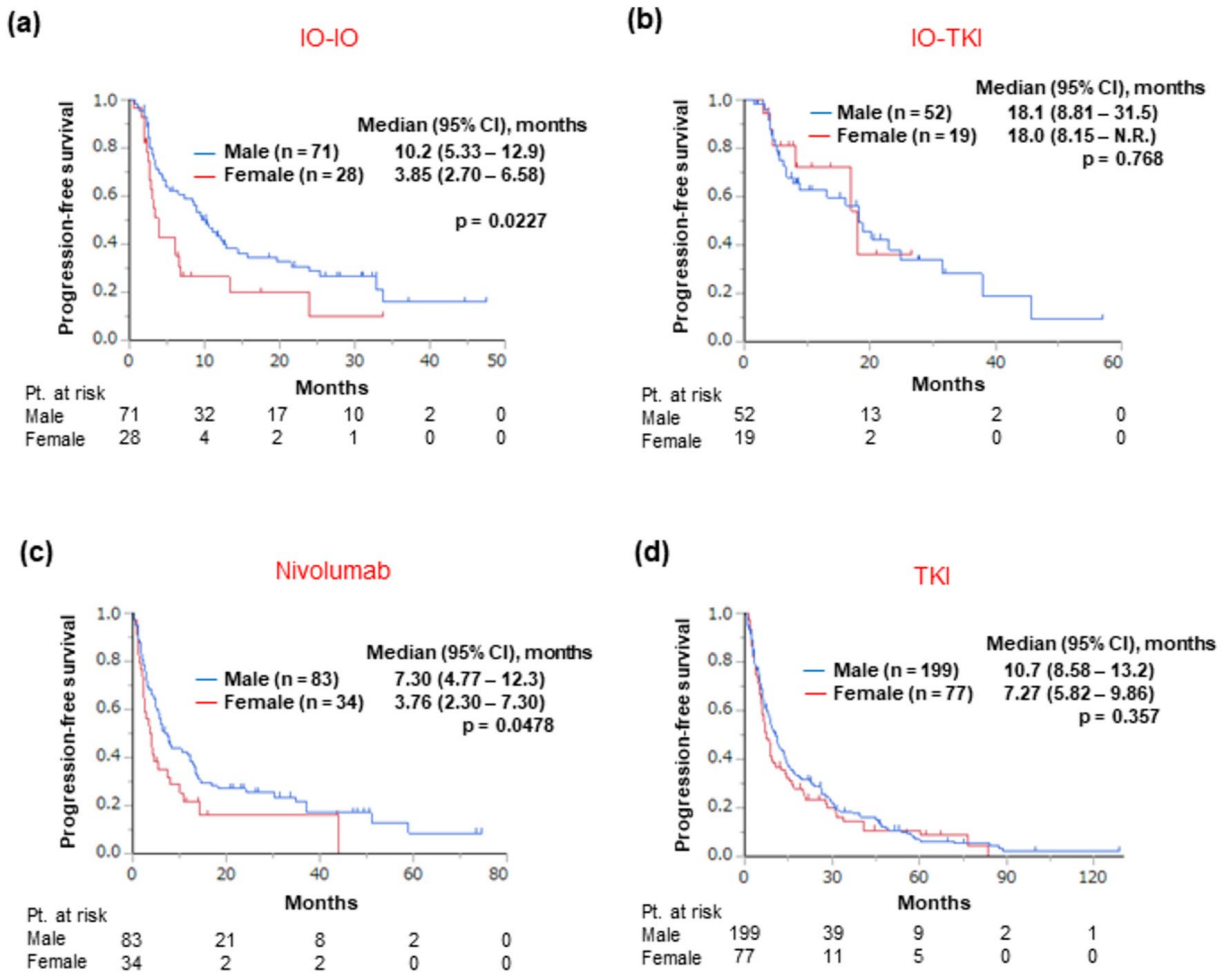
Progression-free survival after treatment initiation across each treatment group stratified by sex, **a** IO-IO treatment group, **b** IO-TKI treatment group, **c** Nivolumab monotherapy group, **d** TKI monotherapy group. *CI* confidence interval, *N.R.* not reached, *Pt*. patient, *IO* immunotherapy, *TKI* tyrosine kinase inhibitor

**Fig. 2:**
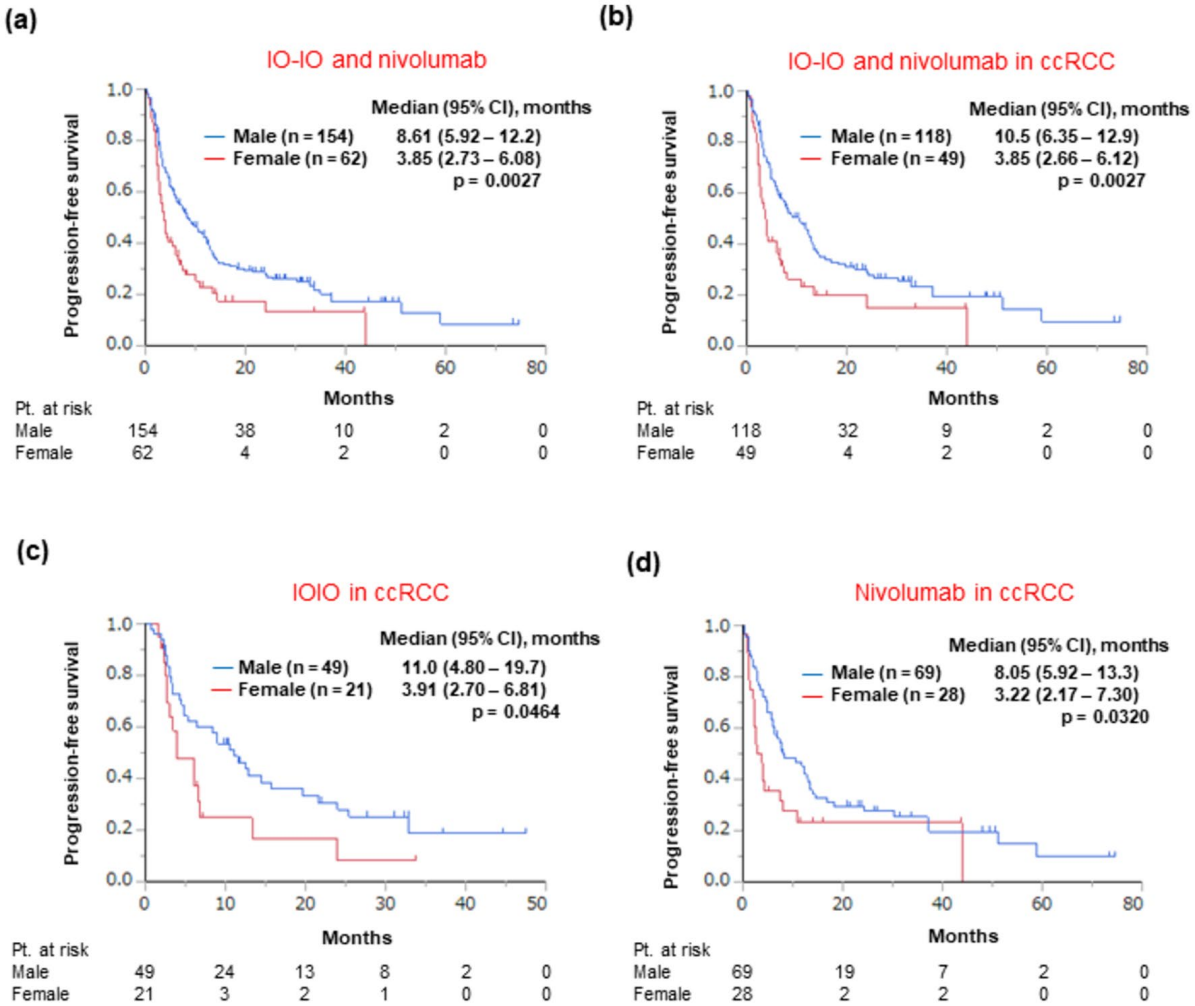
Progression-free survival after treatment initiation in subgroup populations (with IO singlet or doublet therapy) stratified by sex, **a** IO-IO and nivolumab groups, **b** patients with clear-cell renal cell carcinoma treated with IO-IO and nivolumab, **c** patients with clear-cell renal cell carcinoma treated with IO-IO treatment, **d** patients with clear-cell renal cell carcinoma treated with nivolumab. *CI* confidence interval, *Pt* patient, *IO* immunotherapy, *ccRCC* clear cell renal cell carcinoma

During the follow-up period (median, 18.8 months; interquartile range, 8.38–36.5 months), 439 patients experienced disease progression and 286 patients died, respectively. We first analyzed the differences in PFS between the sexes in each treatment group. The PFS was shorter in female patients than in male patients in the IO-IO treatment group (median: 3.85 vs. 10.2 months, respectively, *p* = 0.0227; Fig. 1a) and in the nivolumab monotherapy group (median: 3.76 vs. 7.30 months, respectively, *p* = 0.0478; Fig. 1c). In contrast, PFS was not significantly different between the sexes in the IO-TKI treatment (median: 18.0 vs. 18.1 months, female vs. male patients, respectively, *p* = 0.768; Fig. 1b) or TKI monotherapy group (median: 7.27 vs. 10.7 months, respectively, *p* = 0.357; Fig. 1d). Multivariate analysis further showed that the “sex” was an independent factor of shorter PFS in the IO-IO (hazard ratio [HR]: 1.76, 95% confidence interval [CI]: 1.04–2.98, *p* = 0.0340) (Table 2) and nivolumab monotherapy groups (HR: 1.71, 95% CI: 1.05—2.80, *p* = 0.0322) (Table 3), after adjusting for other covariates identified by the univariate analysis. In a combined cohort of the IO-IO and nivolumab monotherapy groups (i.e., regimens consisting of only ICIs), the PFS was shorter in female patients (median: 3.85 vs. 8.61 months, female vs. male patients, respectively, *p* = 0.0027) (Fig. 2a). In this combined cohort, when patients with clear-cell RCC were analyzed, PFS was again found to be shorter in female patients (median: 3.85 vs. 10.5 months, female vs. male patients, respectively, *p* = 0.0027) (Fig. 2b). When the cohorts was categorized according to various treatment regimens, in patients with clear-cell RCC, PFS was shorter in female patients in the IO-IO (3.91 vs. 11.0 months, female vs. male patients, respectively, *p* = 0.0464) (Fig. 2c) and nivolumab monotherapy groups (median: 3.22 vs. 8.05 months, respectively, *p* = 0.0320) (Fig. 2d). When patients with non-clear-cell RCC treated with either IO-IO or nivolumab monotherapy (n = 38) were analyzed, PFS (median PFS: 3.90 vs. 3.19 months, female vs. male patients, respectively, *p* = 0.972) and OS (median OS: 21.4 vs. 33.9 months, respectively, *p* = 0.721) were not significantly different between the sexes.

**Table 2:** Univariate and multivariate analyses for progression-free survival after IO-IO treatment

	Univariate analysis HR (95% CI)	*p*	Multivariate HR (95% CI)	*p*
Sex Female (ref. male)	1.81 (1.08–3.05)	0.0251	1.76 (1.04–2.98)	0.0340
Age, in years ≥ 65 (ref. < 65)	1.15 (0.72–1.84)	0.564		
Histopathology Clear-cell RCC (ref. non-clear-cell RCC/ unknown)	0.90 (0.53–1.51)	0.679		
IMDC risk Poor (ref. intermediate)	0.98 (0.59–1.63)	0.936		
Lung metastasis Presence (ref. absence)	0.80 (0.49–1.29)	0.363		
Bone metastasis Presence (ref. absence)	1.17 (0.67–2.05)	0.587		
Liver metastasis Presence (ref. absence)	2.12 (1.15–3.89)	0.0155	2.05 (1.11–3.77)	0.0217
Lymph node metastasis Presence (ref. absence)	1.25 (0.76–2.07)	0.377		

*IO* immunotherapy, *HR* hazard ratio, *CI* confidence interval, *ref* reference, *RCC* renal cell carcinoma, *IMDC* international metastatic renal cell carcinoma database consortium

**Table 3:** Univariate and multivariate analyses for progression-free survival after nivolumab treatment

	Univariate analysis HR (95% CI)	*p*	Multivariate HR (95% CI)	*p*
Sex Female (ref. male)	1.57 (1.00–2.46)	0.0499	1.71 (1.05–2.80)	0.0322
Age, in years ≥ 65 (ref. < 65)	0.64 (0.42–0.96)	0.0307	0.64 (0.42–0.98)	0.0383
Histopathology Clear-cell RCC (ref. non-clear-cell RCC/ unknown)	0.58 (0.35–0.97)	0.0364	0.85 (0.49–1.48)	0.566
IMDC risk Favorable Intermediate Poor	0.98 (0.47–2.07) Ref 2.24 (1.42–3.54)	0.0034 0.966 Ref 0.0005	0.90 (0.42–1.92) Ref 1.86 (1.14–3.05)	0.0311 0.778 Ref 0.0133
Treatment line Second (ref. third or more later)	1.11 (0.73–1.69)	0.623		
Lung metastasis Presence (ref. absence)	0.49 (0.32–0.74)	0.0008	0.68 (0.44–1.06)	0.0883
Bone metastasis Presence (ref. absence)	2.50 (1.60–3.89)	< 0.0001	2.08 (1.30–3.33)	0.0022
Liver metastasis Presence (ref. absence)	1.93 (1.13–3.28)	0.0153	2.42 (1.39–4.22)	0.0018
Lymph node metastasis Presence (ref. absence)	1.26 (0.82–1.92)	0.289		

*HR* hazard ratio, *CI* confidence interval, *ref* reference, *RCC* renal cell carcinoma, *IMDC*, international metastatic renal cell carcinoma database consortium

Regarding OS, the survival rate was not significantly different between the sexes in any treatment group (IO-IO: median 49.0 vs. 38.4 months, female vs. male patients, respectively, *p* = 0.606; IO-TKI: median not reached [N.R.] vs. N.R., respectively, *p* = 0.568; nivolumab monotherapy: 39.2 vs. 38.3 months, respectively, *p* = 0.629; TKI monotherapy: 20.4 vs. 26.6 months, respectively, *p* = 0.185) (Supplementary Fig. 1a–1d). In addition, the ORR was not significantly different between sexes in any treatment group (IO-IO: 36% vs. 49%, female vs. male patients, respectively, *p* = 0.353; nivolumab monotherapy: 24% versus 39%, respectively, *p* = 0.262; TKI monotherapy: 25% versus 28%, respectively, *p* = 0.578), except for the IO-TKI group, where the ORR was lower in female patients than in male patients (32% vs. 62%, respectively, *p* = 0.011) (Supplementary Table 2).

These data suggest that the therapeutic effects of ICIs on PFS were lower in female patients than that in male patients, which was uniquely observed when ICIs were used for clear-cell RCC but not with TKI-containing therapy.

### TIIC profile based on the sex

Clinical data indicated that ICIs had inferior therapeutic effects on PFS of female patients compared to male patients, raising the hypothesis that the profile of the immune tumor microenvironment differs between sexes. Therefore, we analyzed the TIIC profiles of 116 RCC tumor samples using flow cytometry and compared them between the sexes. The gating strategy used for flow cytometry is shown in Supplementary Fig. 2. Characteristics of patients whose samples were used for flow cytometry are summarized in Supplementary Table 3. There was no significant difference in age, histopathology, Fuhrman grade, or pathological stage between the sexes (*p* > 0.05).

**Fig. 3:**
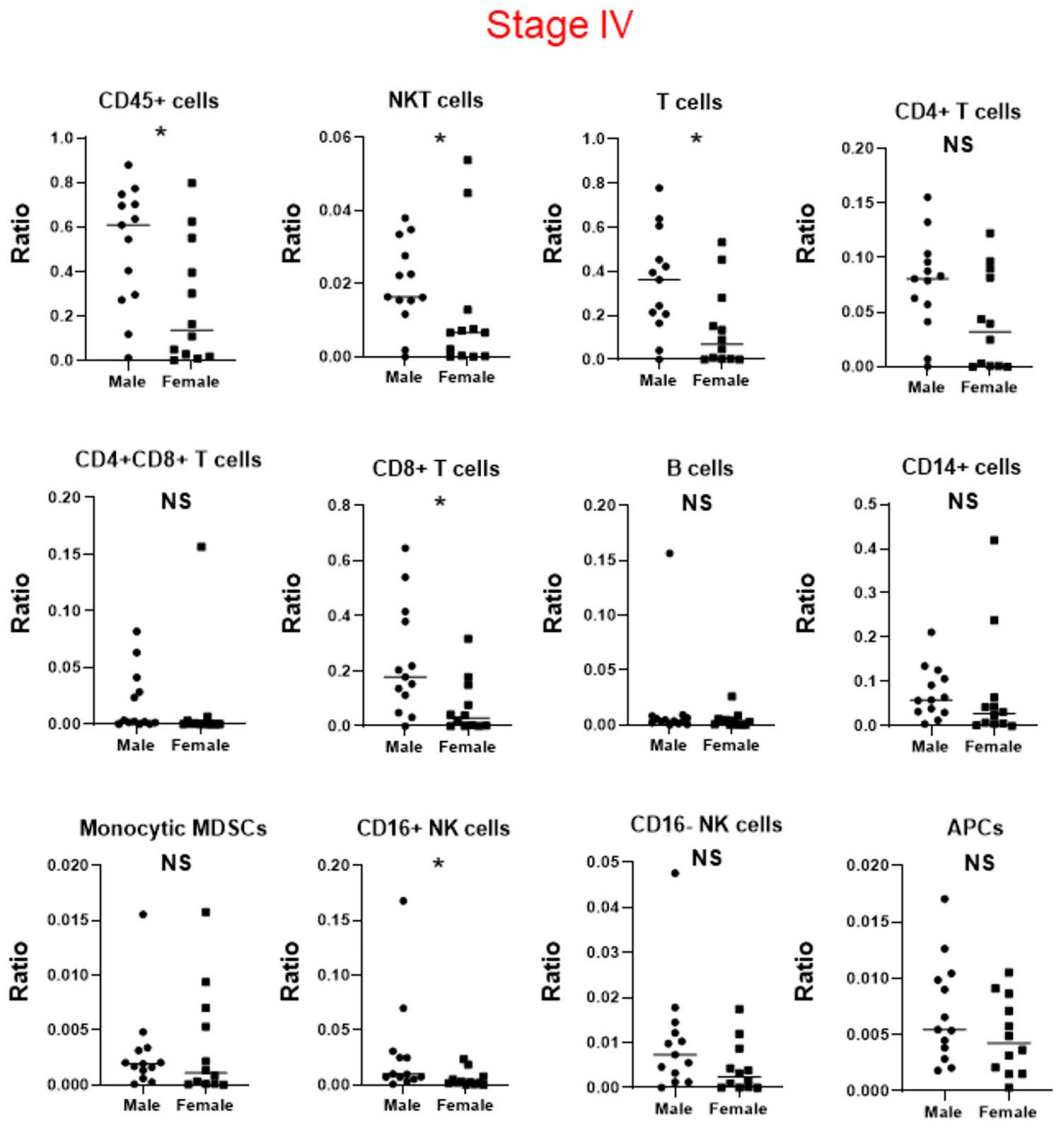
Profiles of tumor-infiltrating immune cells in patients with pathological stage IV renal cell carcinoma stratified by sex, * indicates statistical significance (*p* < 0.05). NS, not significant

In all patients, across all stages of cancer, there was no significant difference in the profile of TIICs between sexes (*p* > 0.05) (Supplementary Fig. 3 and Supplementary Table 4). There was no significant difference between the sexes in patients with stage I and II RCC (*p* > 0.05) (Supplementary Fig. 4 and Supplementary Table 5). In addition, in patients with stage III RCC, there was no significant difference between sexes (*p* > 0.05), except for a higher number of CD4 + T cells/living cells in female than in male patients (*p* = 0.0344) (Supplementary Fig. 5 and Supplementary Table 6). In patients with stage IV RCC, there was a difference in TIIC profiles between sexes, including lower numbers of CD45 + T cells/living cells, NKT cells/living cells, T cells/living cells, CD8 + T cells/living cells, and CD16 + NK cells/living cells in female patients than in male patients (*p* < 0.05) (Fig. 3 and Supplementary Table 7). In patients with stage IV RCC, there was no significant difference in age, histopathology, Fuhrman grade, International Metastatic RCC Database Consortium (IMDC) risk classification, or status of metastatic organ sites between the sexes (*p* > 0.05) (Supplementary Table 8).

We further analyzed sex differences in the profiles of CD45 + cells, NKT cells, T cells, CD8 + T cells, and CD16 + NK cells between patients with localized (stages I—III) and advanced (stage IV) RCC. The number of CD45 + T cells/living cells (*p* = 0.0302), T cells/living cells (*p* = 0.0211), and CD8 + T cells/living cells (*p* = 0.0203) increased in male patients with advanced disease, while it decreased in female patients (*p* = 0.0326, *p* = 0.0427, and *p* = 0.0487, respectively) (Supplementary Fig. 6 and Supplementary Table 9). Furthermore, although the number of NKT cells and CD16 + NK cells was not significantly different in male patients with advanced disease (*p* = 0.478 and *p* = 0.361, respectively), it decreased in female patients (*p* = 0.0096 and *p* = 0.0005, respectively).

Finally, we focused on sex differences in terms of T cells, especially CD8 + T cells, of patients with stage IV RCC (Supplementary Fig. 7). Profiling of tumor-infiltrating T cells based on CCR7 and CD45RA markers that could detect T-cell differentiation [18] revealed that female patients had significantly fewer effector memory (EM)/CD8^+^ T cells (*p* = 0.0054) and significantly more naïve/CD8 + T cells and T-effector memory cells re-expressing CD45RA cells/CD8 + T cells (*p* = 0.0338 and *p* = 0.0084, respectively) (Supplementary Fig. 8 and Supplementary Table 10).

Collectively, these data indicate that the profile of TIICs in RCCs differs between sexes, especially in advanced cases. The decrease in the number of some TIIC populations, particularly that of cytotoxic CD8 + T cells, was potentially associated with the decreased therapeutic effects of ICIs on PFS in female patients compared to male patients.

## Discussion

Using real-world data, we conducted a retrospective analysis to examine the relationship between sex and the efficacy of systemic therapy for RCC across various treatment groups: IO-IO and IO-TKI combination therapies, nivolumab monotherapy, and TKI monotherapy. Furthermore, we explored the disparities in the profiles of TIICs in RCC tumor samples across different sexes. Our study revealed that PFS was shorter in female patients than in male patients receiving nivolumab plus ipilimumab therapy or nivolumab monotherapy. Multivariate analysis further showed that sex was independently associated with PFS in these treatment groups after adjusting for covariates. This significant association between sex and PFS was not observed in TKI-containing treatments, such as the IO-TKI combination therapy and TKI monotherapy. In addition, when analyzed according to histopathological type, a negative association between sex and PFS was uniquely observed in patients with clear-cell RCC. These data indicate that, for patients with clear-cell RCC, a decreased effectiveness on PFS in female patients was specific to ICI-based therapy, and this was not observed in the TKI-containing therapy. Furthermore, TIIC profiling using RCC tumor samples revealed a lower number of some TIIC populations, including that of cytotoxic CD8 + T cells, in female than in male patients, particularly in patients with advanced disease.

The prognostic impact of sex on ICI treatment efficacy for advanced cancers has been debated [11, 12]. There is a scarcity of data on the effect of sex on ICI treatment efficacy in an exclusive cohort of patients with RCC. Real-world data from the IMDC group showed no modification effect of sex in the effectiveness of nivolumab over everolimus as a second-line treatment [19]. Hassler et al. also investigated the impact of sex on the efficacy of ICIs over standard-of-care sunitinib using data from four clinical trials of RCC (CheckMate 025, CheckMate 214, KEYNOTE-426, and JAVELIN Renal 101) [20]. In that study, the therapeutic benefit of ICIs over sunitinib did not differ between the sexes. We previously reported that female patients had inferior PFS with first-line nivolumab plus ipilimumab combination therapy and subsequent nivolumab monotherapy compared with male patients [15]. Thus, the present data reproduced our previous findings using updated data.

We further found that the decreased effectiveness of ICIs in female patients was uniquely observed in a regimen consisting of ICIs alone, but not in TKI-containing regimens. The present TIIC profiling showed potentially lower immune response activity in female patients than in males; furthermore, in cases of advanced disease (i.e., stage IV), the number of CD45 + cells, NKT cells, T cells, CD8 + T cells, and CD16 + NK cells was lower in female patients. These data suggested that female patients harbored the so-called “desert type microenvironment” rather than an “inflamed” or “exhausted type.” Interestingly, the number of CD8 + T cells increased in line with disease progression in male patients, while it decreased in females, presenting a contradictory trend in the CD8 + T cell population between the sexes. Innate and adaptive immunity differ between sexes due to skewed chromosome X inactivation and escape from chromosome X inactivation or the influence of sex steroids, such as estrogens and androgens [21]. According to a recent study, loss of the Y chromosome in tumor cells alters T cell function, promoting T cell exhaustion and sensitizing to PD-1 targeted ICIs [22]. In addition, the Y chromosome gene *KDM5D* drives male-specific metastasis and worse outcomes in colorectal cancer harboring *KRAS* alterations, which activates STAT4 to increase KDM5D, repressing the expression of genes governing cell adhesion and immune recognition [23]. Based on these findings, female patients were expected to have better outcomes and benefit from ICIs, which is inconsistent with our findings. Collectively, it remains unclear how sex affects survival or tumor response to ICIs. Further investigations into profiling of TIICs, focusing on specific T cell subpopulations, such as differentiation, activation, and exhaustion, are needed.

Other possible mechanisms that can explain the impact of sex differences on the effectiveness of ICIs are differences in pharmacokinetics/pharmacodynamics as well as bioavailability and production of anti-drug antibodies (ADAs). However, sex alone has not been identified as a significant factor that clinically affects the drug clearance of ICIs [24]. Additionally, few studies have reported the impact of sex on ADA development against ICIs during cancer treatment. For patients with benign disease, sex has a possible effect on ADA production with some anti-TNF monoclonal antibodies. For example, male patients had higher serum ADA levels than those of female patients who were administered infliximab; however, such sex differences were not observed with adalimumab administered to patients with inflammatory bowel disease [25]. For patients with rheumatoid arthritis, ADA development against infliximab more commonly occurred in female patients (26). Therefore, further studies should be performed to investigate sex differences in pharmacokinetics/pharmacodynamics and ADA development against monoclonal antibodies.

This study is subject to certain limitations. First, the retrospective nature of the study coupled with a small sample size, may have potentially introduced a selection bias that could influence the findings. In addition, the present cohort exhibited strong heterogeneity in terms of systemic therapy classes and patient characteristics, which potentially affected the findings. Second, the insufficient follow-up period complicated the interpretation of the OS data. Future, large-scale, long-term studies are needed to validate our findings. Third, there exist undetected confounding factors between the sexes, such as past medical history or habit history (e.g., smoking or alcohol consumption), as well as hormone status, which might potentially affect the outcome data. Therefore, future studies conducted using more comprehensive data and statistical modelling (e.g. multivariable regression analysis and matched groups) to control for these potentially confounding factors are needed.

In conclusion, this retrospective study using real-world data showed inferior PFS after ICI treatment, including nivolumab plus ipilimumab combination therapy and nivolumab monotherapy, in female patients with advanced RCC compared to males. This significant association between sex and PFS was uniquely observed with ICI-based therapy for patients with clear-cell RCC, but not with TKI-containing therapy. The profiles of TIICs in RCC tumor samples revealed a reduced populations for some TIICs, particular that of cytotoxic CD8 + T cells, in female patients compared to male patients with advanced disease. These data suggest that a different profile of the immune tumor microenvironment between the sexes might induce different effects of ICIs in patients with RCC.

## Supplementary Information

Below is the link to the electronic supplementary material.

Supplementary file1 (XLSX 35 KB)

Supplementary file2 (TIF 111 KB)

Supplementary file3 (TIF 333 KB)

Supplementary file4 (TIF 114 KB)

Supplementary file5 (TIF 110 KB)

Supplementary file6 (TIF 107 KB)

Supplementary file7 (TIF 95 KB)

Supplementary file8 (TIF 198 KB)

Supplementary file9 (TIF 84 KB)

## Data Availability

No datasets were generated or analysed during the current study.
