# Supplementary figures and images for: Sex differences in immunotherapy outcomes and tumor-infiltrating immune cell profiles in patients with advanced renal cell carcinoma
Source: Cancer Immunol Immunother. 2025 Jan 3;74(2):51. doi: 10.1007/s00262-024-03876-2 (PMC11699158; doi:10.1007/s00262-024-03876-2)

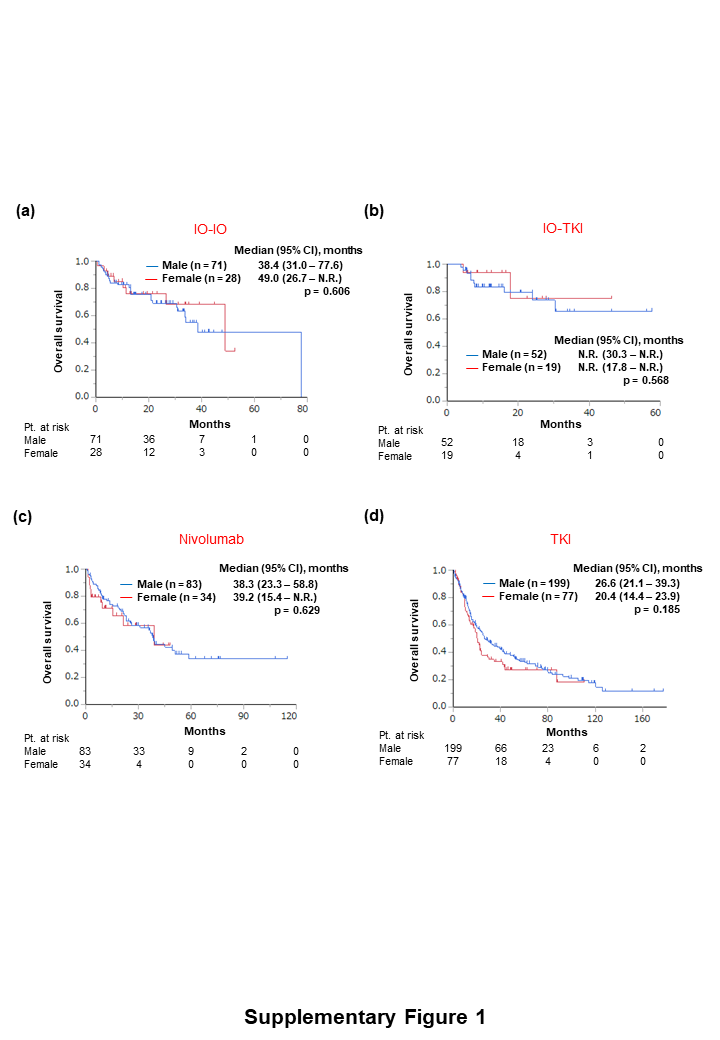

Supplement: Supplementary file 2 — Supplementary file2 (TIF 111 KB) [file 262_2024_3876_MOESM2_ESM.tif]

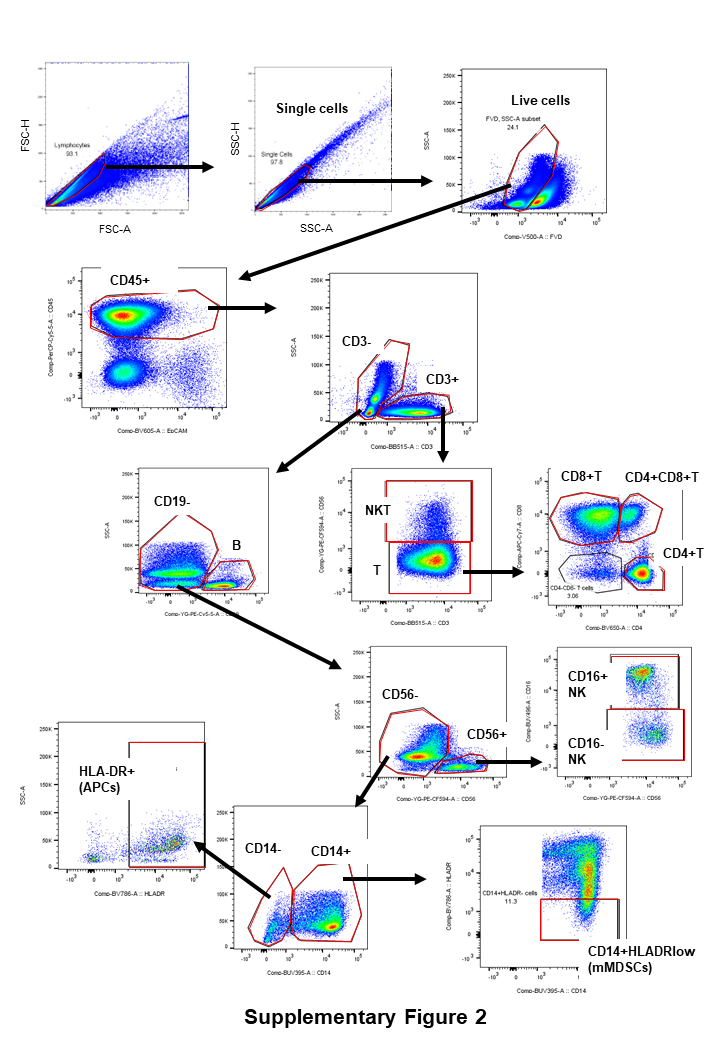

Supplement: Supplementary file 3 — Supplementary file3 (TIF 333 KB) [file 262_2024_3876_MOESM3_ESM.tif]

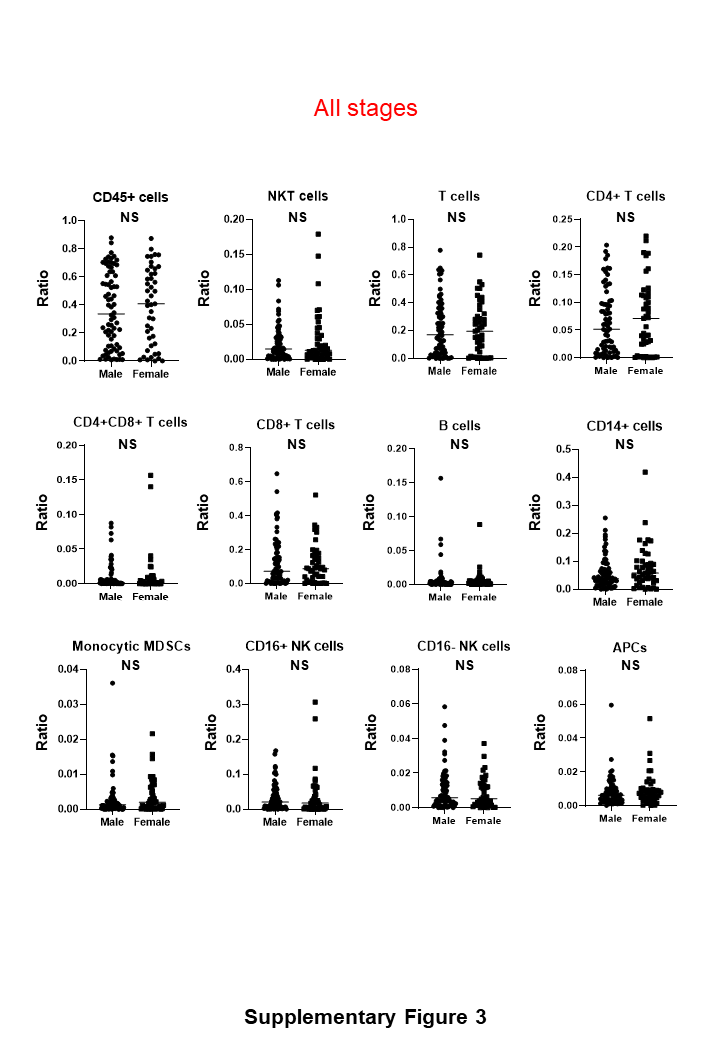

Supplement: Supplementary file 4 — Supplementary file4 (TIF 114 KB) [file 262_2024_3876_MOESM4_ESM.tif]

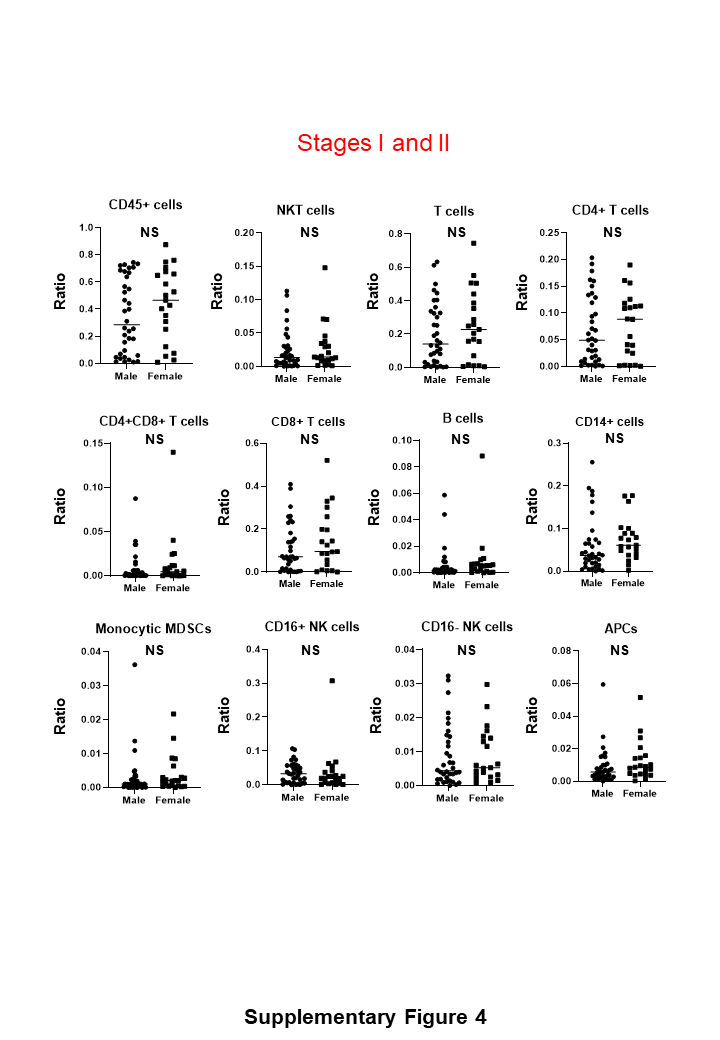

Supplement: Supplementary file 5 — Supplementary file5 (TIF 110 KB) [file 262_2024_3876_MOESM5_ESM.tif]

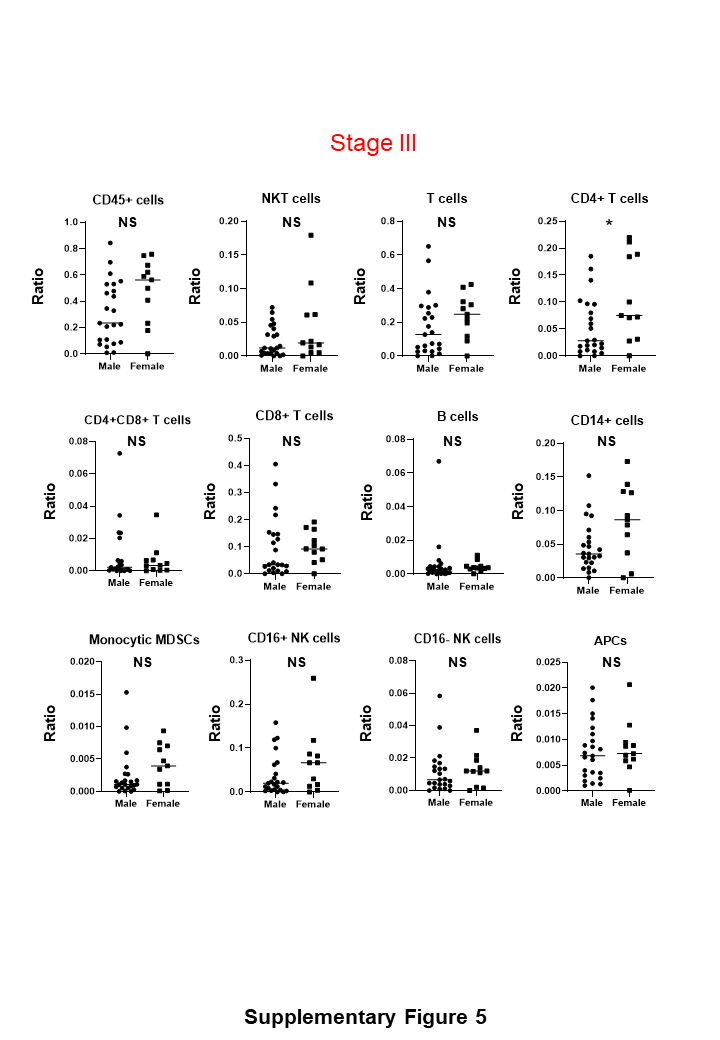

Supplement: Supplementary file 6 — Supplementary file6 (TIF 107 KB) [file 262_2024_3876_MOESM6_ESM.tif]

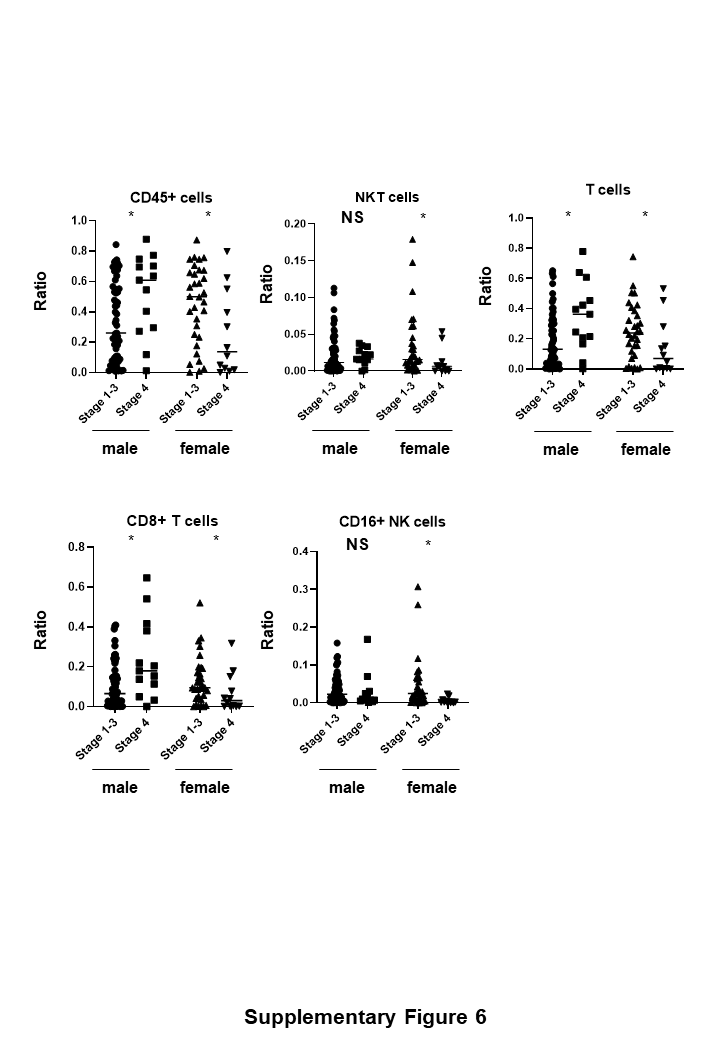

Supplement: Supplementary file 7 — Supplementary file7 (TIF 95 KB) [file 262_2024_3876_MOESM7_ESM.tif]

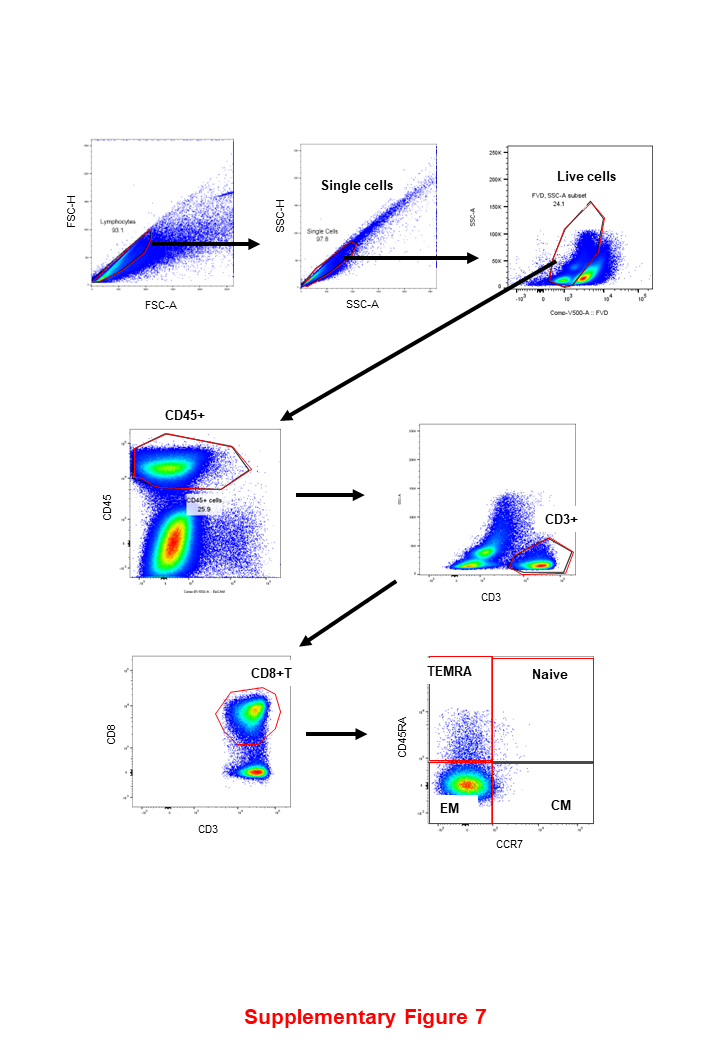

Supplement: Supplementary file 8 — Supplementary file8 (TIF 198 KB) [file 262_2024_3876_MOESM8_ESM.tif]

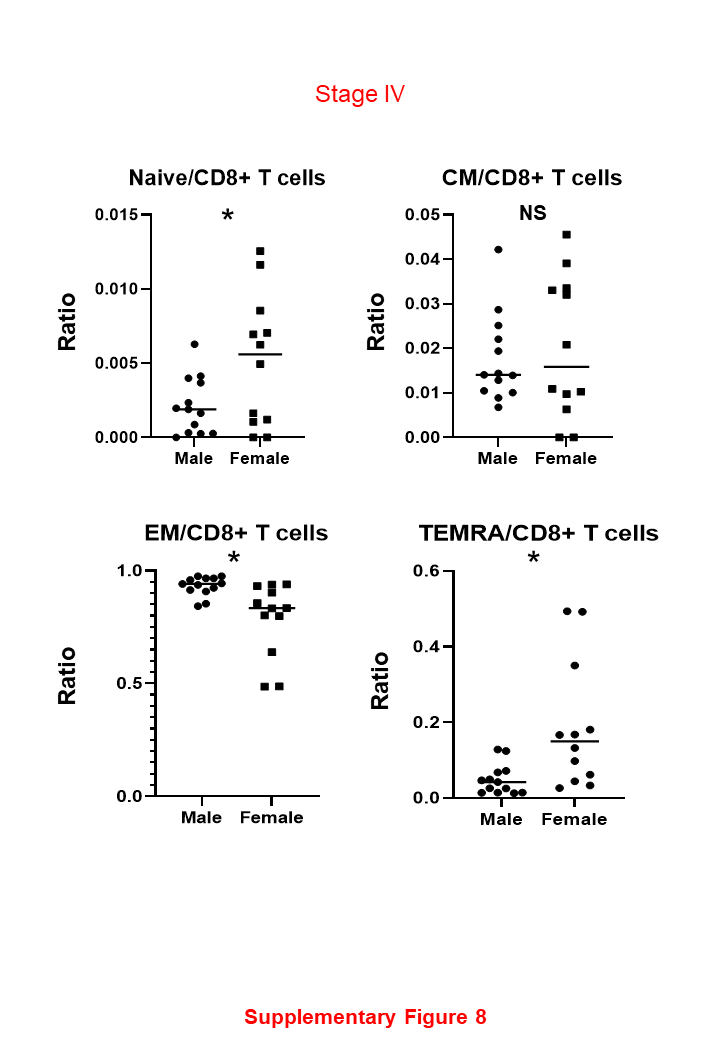

Supplement: Supplementary file 9 — Supplementary file9 (TIF 84 KB) [file 262_2024_3876_MOESM9_ESM.tif]
